# Dietary interventions to support and improve sleep disturbances and insomnia disorder in menopause: From bench to bedside

**DOI:** 10.1177/20533691251350518

**Published:** 2025-07-02

**Authors:** Rosie H Musgrave, Sara Nowakowski, Tamlyn J Watermeyer, Emily J Arentson-Lantz, Greg J Elder

**Affiliations:** 1 Northumbria Centre for Sleep Research, Department of Psychology, 5995 Northumbria University, Newcastle Upon Tyne, UK; 2 Department of Medicine, 3989 Baylor College of Medicine, Houston, TX, USA; 3 Faculty of Health & Life Sciences, 5995 Northumbria University, Newcastle Upon Tyne, UK; 4 Department of Nutrition Sciences and Health Behavior, 12338 The University of Texas Medical Branch, Galveston, TX, USA

**Keywords:** Sleep, insomnia, dietary intervention, menopause

## Abstract

Sleep is essential for maintaining physical and psychological health, and also cognitive health (referred to as ‘brain health’). However, the transition to menopause has a direct impact upon sleep. Sleep disturbances are reported by approximately 40%–60% of menopausal women, and insomnia disorder is also prevalent. Diet-based interventions could potentially be used to improve subjective sleep quality in this population, and although there are several promising interventions which have been used in other groups that could be trialled, the evidence base is currently lacking. One particularly promising area for future research is that these interventions might be used alongside, or instead of, established treatments for insomnia disorder. This may also help to prevent the development of longer-term insomnia disorder. Future studies should use well-controlled, adequately powered, clinical trial designs to assess the effectiveness of dietary interventions in improving subjective sleep quality, treating insomnia disorder, and preventing acute sleep disturbances from progressing to chronic insomnia. Given the strong association between sleep and neurodegeneration, optimizing sleep in menopausal women, using targeted diet-based strategies, may have significant implications for brain health.

## Introduction

Sleep is essential for physical and psychological health and well-being. Conversely, disrupted sleep, or sleep loss, directly contributes to multiple negative health outcomes^1,2^ including type 2 diabetes, hypertension, cardiovascular disease and obesity.^3–5^ Sleep is a complex physiological process which can be measured and quantified using subjective and/or objective methods: subjective methods include questionnaires, sleep diaries and estimating sleep duration; objective methods include actigraphy (accelerometery) or polysomnography (PSG).^6^ Although subjective and objective sleep changes as a normal function of ageing, the menopausal transition in women has a particularly negative effect on sleep.^6,7^ Menopause is a natural physiological event which typically occurs at approximately 50 years of age in Western women, and refers to the permanent cessation of menstrual periods due to ovarian follicular depletion occurring at the same time as alterations to the ovarian hormones estrogen and progresterone.^8,9^

### Sleep disturbances and insomnia disorder in menopause

Sleep disturbance is a core menopause symptom^7^ that is reported by 40%–60% of menopausal women.^10^ Menopause-related sleep disturbances are predominantly subjective, and include problems initiating and maintaining sleep, accompanied by the daytime symptom of excessive sleepiness.^7^ Menopausal vasomotor symptoms, including nocturnal hot flashes or night sweats, can negatively affect sleep.^11^

Insomnia disorder, the clinical diagnosis defined as disturbed sleep associated with distress and/or impairment, is particularly common in menopause with a prevalence of 38%–60% in peri- and postmenopausal women.^8^ There are well-established sex differences, as insomnia is twice as common in women than men,^12^ and the prevalence increases around the time of menopause onset.^13^ This is important because the prevalence of insomnia symptoms is typically higher than the prevalence of insomnia disorder: approximately 6%–10% of the population within industrialised societies meet diagnostic criteria for insomnia, but up to 48% of the postmenopausal population report insomnia symptoms.^14,15^ Although less is known about the impact of menopause upon objective sleep, some evidence suggests objective sleep quality (sleep efficiency) is impaired and nocturnal wake time is increased relative to non-menopausal women.^16^ Physiological hyperarousal during non-rapid eye movement sleep independent of self-reported hot flashes has also been reported by women transitioning to menopause.^17^

### Consequences of disturbed sleep and insomnia disorder: Implications for brain health

Globally, the number of postmenopausal women will total 1.2 billion women by 2030.^11^ The growing number of ageing women with associated with the menopause transition are at risk of negative physical, cognitive and psychological health outcomes^1,2^ associated with insomnia disorder, contributing to a significant economic and societal burden.^18–21^

Both experimental and epidemiological studies indicate sleep represents an important modifiable risk factor for the prevention of dementia.^22^ Individuals who report sleep disturbances have a greater subsequent risk of developing neurodegenerative dementia, compared to individuals who do not report sleep disturbances.^23^ Sleep has a mechanistic role in neurodegeneration, as experimental sleep deprivation increases β-amyloid, which is directly involved in subsequent dementia development.^24^ Additionally, sleep fragmentation and insufficient sleep duration are associated with reduced hippocampal volume, altered brain network functional connectivity and increased β-amyloid accumulation^25^; these factors have a role in the pathogenesis of Alzheimer’s dementia (AD) and other neurodegenerative disorders. Women are at greater risk for AD following the menopausal transition, which is attributed in part to declining sex hormone levels. The loss of the protective effect of estrogen on the brain can also potentially affect objective sleep architecture, and circadian activity, by interacting with melatonin and orexin signalling pathways, which are relevant to sleep and wake.^26^ These neurobiological disruptions not only contribute to cognitive decline but also exacerbate mood disorders. The menopausal transition is associated with an increased risk of depression and anxiety which is linked to fluctuations in estrogen affecting other neurotransmitters involved in sleep/wake regulation, including serotonin (5-HT) and γ-aminobutyric acid (GABA).^27^ This is important as there are well-established bi-directional relationships between anxiety, depression and insomnia.^28^ Therefore, interventions that maintain sleep in menopausal women may also maintain good brain health.

### Dietary interventions for the treatment of sleep disturbances and insomnia disorder in menopause

Diet is a well-established modifiable health behaviour to mitigate the risk of chronic disease. Emerging evidence from epidemiological studies indicate a link between overall diet quality indices and aspects of subjective sleep quality, or continuity (the timing and distribution of sleep), as well as the risk of sleep disorders.^29,30^ The consumption of ultra-processed foods is inversely related to sleep outcomes; conversely, adherence to a Mediterranean-based diet, which is typically rich in vegetables, fruits, whole grains, nuts and lean protein, is the dietary pattern with the strongest evidence of improving sleep quality.^31,32^ Plant-based foods provide an abundance of dietary bioactives, such as polyphenols, which may provide one of the biological links between diet and sleep due to their neuromodulating effect.^33,34^ Additionally, consuming a higher proportion of energy intake from protein and relatively lower proportion from carbohydrates and fat is associated with increased sleep duration, lower sleep latency and greater sleep efficiency.^35^ However, future work is necessary to establish mechanistic links between dietary quality and sleep as well as optimise ‘dose’ recommendations, especially for populations at risk of poor sleep quality, such as menopausal women.

In addition to considering overall diet quality, specific food items that are sources of neuromodulating bioactives can improve aspects of subjective sleep quality or continuity (i.e. the timing and distribution of sleep).^36^ Data from clinical trials demonstrate walnuts and tart cherry juice promote melatonin levels which translate to some modest improvements in sleep outcomes; similarly, foods that are rich in tryptophan, such as dairy, are positively associated with sleep outcomes.^37^ This is biologically plausible as tryptophan is an amino acid that is converted to sleep-promoting, melatonin and serotonin. A recent systematic review of food-based interventions the treatment of sleep disturbances in menopausal women indicated that although the majority of studies were of poor quality, nutritional interventions did appear to benefit subjective sleep.^36^ While beyond the scope of this brief review, diet quality and specific dietary bioactives also play an important role in supporting brain health. Similar to sleep, consuming fruits, vegetables and whole grains provide polyphenols and antioxidants that are protective of neurodegeneration, as seen in AD and other neurodegenerative dementias.^38–40^

#### Sleep disturbances and menopause vasomotor symptoms

An additional consideration of improve sleep outcomes specific to the menopausal transition is interventions that alleviate the vasomotor symptoms associated with poor sleep outcomes in menopausal women.^11,41^ In particular, a recent review has indicated that soy/soybean-based products are worthy of further investigation, due to the potential mechanistic action of the isoflavone content in reducing reduce vasomotor symptoms; black cohosh may also benefit sleep by acting on relevant sleep/wake neurotransmitters.^36^

#### Insomnia disorder

The first-line treatment for insomnia disorder is cognitive behavioural therapy for insomnia (CBT-I), which is highly effective in many different populations,^28^ including postmenopausal women.^11^ Alternatively, pharmacological treatment (benzodiazepines (BZs) or benzodiazepine receptor agonist (BZRAs)) can also be used. However, both CBT-I and BZRA treatments have potential limitations, which make diet-based interventions worthy of further study. With regards to CBT-I, although it is highly effective for insomnia, the main limitations of this intervention are that there are very few qualified CBT-I practitioners as well as high levels of attrition from CBT-I programmes.^42^ Although pharmacological treatments are effective for insomnia in the short term, they have side effects (e.g. subsequent drowsiness and dependency) are linked to negative health outcomes and are unsuitable for certain groups of people.^6^ Nutritional interventions may be particularly attractive for these individuals. There is some evidence which indicates that healthy diets are associated with a lower prevalence of insomnia, potentially due to common underlying mechanisms.^43^

However, perhaps surprisingly, very little research has specifically investigated the effectiveness of nutritional interventions for insomnia in menopause. Only four studies have done this to date, where insomnia has been defined as such on the basis of self-report measures and not using recognized diagnostic criteria such as the Diagnostic and Statistical Manual of Mental Disorders (DSM-5) or the International Classification of Sleep Disorders (ICSD-3).^36^ Specific nutritional interventions that warrant testing include isoflavones, melatonin and proanthocyanin, since a recent systematic review has indicated these studies appeared to benefit women with self-reported insomnia symptoms.^36^ However, there are important methodological issues with these studies, including a lack of clinical trial pre-registration and inappropriate control groups or comparator conditions.^36^ As mentioned previously, a recent review suggested that isoflavones, soy/soybean-based interventions and black cohosh might improve subjective sleep^36^; given that insomnia disorder is subjective in nature, a starting point would be to assess these interventions in menopausal women with a diagnosis of insomnia disorder, using well-controlled clinical trial designs. However, interventions with a sound mechanistic basis for benefiting sleep are needed.

#### Chrononutrition

Finally, the timing of nutrition may also have an impact upon sleep (i.e. ‘chrononutrition’^44^) and could potentially be used as an intervention in itself. Chrononutrition refers to food intake which is optimally aligned with the body’s circadian rhythm,^44^ where circadian rhythms refer to the normal approximately 24-h oscillatory patterns of physiological and behavioural processes, which have an impact upon the timing and quality of sleep.^45^ Less desirable dietary patterns including fewer main meals, frequent snacks and late-night eating have an inverse relationship with sleep. The menopausal transition may alter circadian rhythmicity, as there is some indication that after menopause, there is a specific shift towards having an earlier sleep and wake time, potentially due to menopause-related hormonal changes.^46^ Chrononutrition is a relatively recent research area and to date, few studies have been conducted.^44^

#### Treatment of acute sleep disturbances/acute insomnia

Diet-based interventions might be particularly useful for the treatment of acute menopause-related sleep disturbances. This could also have implications for successfully preventing short-term sleep disturbances, or the short-term sleep disorder of acute insomnia, from progressing to insomnia disorder, in menopausal women. On the basis of theoretical models of insomnia, menopausal symptoms could act as a precipitating factor, and trigger a short-term sleep disturbance.^11^ This is important because early interventions are needed to prevent acute insomnia from progressing to longer term insomnia disorder, and non-pharmacological treatments are likely to be particularly suitable as an early intervention.^47^

### Research priorities

Well-designed adequately powered studies should assess if diet-based interventions can:

1. Improve subjective sleep quality and improve the subjective sleep of menopausal women with short-term sleep disturbances.
2. Treat insomnia disorder, as a potential alternative or addition to effective methods of treatment such as CBT-I, or alternatives to potentially problematic drug treatments such as BZ/BZRA agents.
3. Specifically treat menopausal women with an acute sleep disturbance, or acute insomnia, to prevent the development of longer-term insomnia disorder.
4. Target menopausal symptoms which negatively affect sleep, as this is likely to benefit sleep and mental health.
5. Benefit brain health using suitable outcome measures in order to assess if targeting sleep can maintain good brain health.

## Conclusion

Sleep disturbances, including the clinical sleep problem of insomnia disorder, are highly prevalent in menopausal women. These may be due to changing levels of estrogen affecting sleep/wake modulatory neurotransmitters, the occurrence of menopausal symptoms negatively affecting sleep, or comorbidities including depression. Dietary interventions offer a promising non-pharmacological route by which sleep could be improved. Future research should use well-controlled clinical trials to determine the effectiveness of dietary interventions in treating both acute sleep disturbances and insomnia disorder in menopausal women.

## Data Availability

This is not applicable to this article as no datasets were generated or analysed during the current study.*
